# HGF/c-Met signaling promotes the migration and proliferation of deer antler MSCs

**DOI:** 10.1038/s41598-023-38116-7

**Published:** 2023-07-10

**Authors:** Miao Wang, Chuan Lin, Xiaodong Jia, Di Fang, Qinhua Gao, Chunmei Han

**Affiliations:** 1 College of Animal Science and Technology, Tarim University, Alar, 843300 China; 2 Key Laboratory of Tarim Animal Husbandry Science and Technology, Xinjiang Production and Construction Corps, Alar, 843300 China; 3 College of Life Science and Technology, Tarim University, Alar, 843300 China; 4 Key Laboratory of Protection and Utilization of Biological Resources in Tarim Basin, Xinjiang Production and Construction Corps, Alar, 843300 China

**Keywords:** Cell biology, Stem cells

## Abstract

The complete regeneration of deer antlers is based on the proliferation and differentiation of stem cells. Mesenchymal stem cells (MSCs) of antlers have an important role in antler regeneration and rapid growth and development. HGF is mainly synthesized and secreted by mesenchymal cells. After binding to its receptor c-Met, which transduces signals into cells to stimulate cell proliferation and migration in various organs to promote tissue morphogenesis and angiogenesis. However, the role and mechanism of the HGF/c-Met signaling pathway on antler MSCs are still unclear. In this study, we established antler MSCs with overexpression and interference of HGF gene by lentivirus and small interference RNA, observed the effect of HGF/c-Met signal pathway on the proliferation and migration of antler MSCs, and detected the expression of downstream related signal pathway genes, to explore the mechanism of HGF/c-MET signal pathway on the proliferation and migration of antler MSCs. The results showed that the HGF/c-Met signal affects the expression of *RAS*, *ERK* and *MEK* genes, regulates the proliferation of pilose antler MSCs through Ras/Raf, MEK/ERK pathway, affects the expression of *Gab1*, *Grb2*, *AKT* and *PI3K* genes, and regulates the migration of MSCs of pilose antler through Gab1/Grb2 and PI3K/AKT pathway.

## Introduction

Tarim red deer, also known as *Cervus elaphus yarkandensis*, is the only subspecies of red deer endemic to Xinjiang, China. Along with some other male subspecies, Tarim red deer can grow antlers once a year (the growth rate of developing antlers can reach up to 2.75 cm/day^1^). Antlers are mammalian organs that can fully regenerate and are an important animal product in Asia^2–4^. Antler regeneration is a stem cell-based epimorphic process; antlerogenic periosteal cells (APCs) are responsible for the pedicle and first-time antler development, while pedicle periosteal cells (PPCs), which are directly derived from APCs, are responsible for annual antler regeneration. Rapid antler growth is accomplished by reserve mesenchyme cells (RMCs), also known as antler MSCs^2–4^. Yet, the exact mechanism of antler regeneration is still not fully understood.

A study found that the proliferation ability of Antler stem cells (AnSCs) is significantly stronger than that of human placenta mesenchymal stem cells in vitro^5^. Furthermore, an animal experiment reported a higher skin wound healing in rats treated with antler MSCs than of human umbilical cord mesenchymal stem cells and rat bone marrow mesenchymal stem cells, further indicating that AnSCs are a class of stem cells with great clinical applications^6^.

Hepatocyte growth factor (HGF) is primarily synthesized and secreted by mesenchymal cells. HGF binds to its specific receptor c-Met (a receptor tyrosine kinase) to transmit signals into cells to recruit downstream signal molecules, thus regulating cell growth, motility, morphogenesis, and regeneration of damaged organs^7^. Liu et al. used different concentrations of HGF for serum-free culture of rat BMSCs and found that the migration ability of MSCs increased with the increase of HGF concentration^8^; Furthermore, Chang et al. suggested that the activation of the HGF/c-Met signal pathway is the main pathway of alveolar type II epithelial cell proliferation induced by ultrafine carbon black^9^. However, studies by Forte et al. showed that the complete Iscove’s modified Dulbecco’s medium containing 20 ng/mL HGF could only promote the migration of mouse BMSCs, but not the proliferation^10^. Also, Wen et al. showed that in an osteogenic differentiation medium, low concentrations of HGF (20 ng/mL) preferentially promoted osteogenic differentiation of rabbit BMSCs, whereas high concentrations of HGF (100 ng/mL) significantly induced cell proliferation^11^.

HGF and c-Met can regulate cell activities through RAS, MAPK, PI3K/AKT and other signaling pathways, including proliferation, survival, movement, invasion and angiogenesis^12,13^. In the liver regeneration phase, HGF promotes liver regeneration by activating RAS, RAF^14^, ERK^15^, and MEK^16^ with HGF/c-Met to initiate cell proliferation. The PI3K/AKT pathway is involved in various cellular processes, including cell cycle entry, cell growth, cell survival and cell migration^17^, and has been repeatedly verified in antler regeneration of red deer and sika deer by promoting mesenchymal stem cell migration^18–20^. Grb2 and Gab1 promote the migration of mouse MSCs through HGF/c-Met^21^.

Antler MSCs are a class of newly discovered stem cells^3^. The mechanism of the effect of HGF/c-Met signaling and downstream pathways on the proliferation and migration of antler MSCs remains unclear. In our previous study, the expression of *HGF* and *c-Met* genes in the damaged and repaired antler and healthy antler tissues was detected by qRT-PCR and immunohistochemistry. The results showed that the relative expression of *HGF* and *c-Met* genes in the damaged antler tissues was significantly higher than that in the healthy antler tissues^22^. Thus, in this study, we further investigated the regulatory mechanism of the proliferation and migration of antler MSCs. These data can provide a basis for analyzing the regulatory mechanism of antler growth, development and regeneration and provides an important theoretical basis for the clinical application of antler MSCs.

## Material and methods

### Cell culture

Antler MSCs cells were a kind gift from Qinghua Gao Laboratory. The cell culture scheme was performed as previously described (Supplementary Method)^23^.

### Construction of the HGF expression vector

In order to determine the effect of HGF on the migration and proliferation of antler MSCs, the lentivirus overexpression vector of the HGF gene was constructed and then sent to Tsingke Biotechnology Co., Ltd. for lentivirus packaging. Tarim red deer antler MSCs were transfected with lentivirus according to the manufacturer’s instructions.

### siRNA transfection

Three pairs of HGF siRNAs (siRNA1, siRNA2, and siRNA3) were selected for transfection experiments. The siRNA oligonucleotides were synthesized by Tsingke Biotechnology Co., Ltd. Transfection was performed for 48 h using Lipofectamine 2000 (Invitrogen, No. 1070962) transfection reagent according to the manufacturer's protocol. The interference efficiency was detected by qPCR and Western blot. siRNA1 showed the highest effect, and thus was selected for further experiments. The sequence was the following: Sense chain 5′-GAUCAACUCAGAUGGUCUATT-3′; Antisense chain 5′-UAGACCAUCUGAGUUGAUCTT-3′.

### RNA extraction and quantitative real time-PCR (qRT-PCR)

Total RNA was extracted from 10^7^ Tarim red deer antler MSCs with 1 mL of Trizol (Invitrogen) reagent, after which RNA was reverse transcribed into cDNA using HyperScript III RT SuperMix (EnzyArtisan). qRT-PCR was carried out using the Fast SYBR Green Master Mix Bulk Pack (Invitrogen). The primer sequences are shown in Table 1, and they were synthesized by the Sangon Biotech (Shanghai) Co., Ltd. The GAPDH gene was used as an internal reference, and the following cycling condition: 95 °C for 3 min, 40 cycles of 95 °C for 5 s, 60 °C for 10 s, and 72 °C for 15 s. For each sample, three biological replicates were used. The 2^−ΔΔCt^ analysis method was used to calculate the gene expression level^24^. The ratio of each gene expression was obtained by comparing the gene expression levels in different treatment samples.

**Table 1:** Primer sequences used for qRT-PCR.

Gene	Species	Forward primer	Reverse primer
HGF	Deer	CCAATGTGCCAATAGATG	TTAGTGATAGATACCGTTCC
c-Met	Bos	TCATCAACTTCTTCGTGGGC	CTTTCAAAGGCGTGGACATACT
RAS	Deer	CCAGTGGAGGATGACGAG	CCCATAGGCAGGAGTGAA
MEK	Bos	CGAAAGGCAAGAAGCGAAAC	TCCACGATGGGCTCCAGGTC
ERK	Bos	AGACGCAACACCTCAGCA	GCCAAGCCAAAGTCACAGA
Grb2	Deer	AGAATGGAAGCCATCGCCAA	GGATGAAGCCGTCTTTCCCA
Gab1	Bos	AGAGCGATGCTGAGTAAA	ATGGCAGATTGTGAAGGT
AKT	Deer	CGCACCGCTCCAAAGAAA	ACGGCTGCACGTAGACACC
P13K	Bos	GCAGACTGGAGGGAGGTGA	TCCGCAAGGTCAAAGTGTAA
GAPDH	Bos	TGTTTGTGATGGGCGTGAACCA	ATGGCGTGGACAGTGGTCATAA

### Western blot

The protein was extracted from 10^6^ Tarim red deer antler MSCs using Radio Immunoprecipitation Assay Lysis buffer (RIPA) (Solarbio) lysate. The total protein concentration was measured using a Pierce BCA Assay kit (Beijing Dingguo Changsheng Biotechnology Co., Ltd.) and denatured by heating. The protein samples (50–100 μg) were assessed by 8% sodium dodecyl sulfate–polyacrylamide gel electrophoresis (SDS-PAGE), wet transferred to polyvinylidene fluoride (PVDF) (Pall), and incubated with primary antibody against HGF (1:3000, Affinity) and GAPDH (1:10,000, Epizyme Biotech) in blocking buffer (5 g skim milk powder, 100 mL TBST buffer (0.05% Tween 20, 1.65 mL + TBS, 700 mL, pH 7.5)) at 4 °C overnight. Next, after being washed by TBST three times and incubated with HRP-conjugated affinipure goat anti-rabbit IgG (Affinity) for 2 h at room temperature, immunoreactivity was visualized using an ECL (Pierce) detection system secondary antibody.

### Cell viability detection

Cell viability was detected by cell counting kit-8 (CCK-8, Beyotime). Cells were seeded at a density of 100 cells/μL into 100 μL per well in a 96-well plate and cultured for 6 h, 12 h, 24 h, 48 h, 72 h, 96 h, and 120 h. After each time point, 10 μL sterile CCK-8 reagent was added to each well. After 1.5 h of incubation, an enzyme labeling instrument measured the absorbance of 450 nm. Cell viabilities at the individual time were normalized to those at 6 h.

### EdU assay

The 5-ethynyl-20-deoxyuridine (EdU) incorporation assay was performed with an EdU assay kit (Ribobio) according to the manufacturer’s instructions. Cells were seeded into 96-well plates at 5 × 10^3^ cells per well for 24–48 h and then exposed to 10 μM EdU for 3 h at 37 °C. Subsequently, the cells were fixed with 4% paraformaldehyde and then permeabilized with 0.5% Triton X-100. Finally, the cells were reacted with 100 μL of 1 × Apollo ® reaction cocktail for 30 min, followed by incubation with 100 μL of Hoechst 33,342 (5 μg/mL). The images were taken with a Nikon T1-SM inverted microscope. The percentage of EdU-positive cells was calculated by dividing the number of EdU-positive cells by the number of Hoechst-stained cells.

### Wound healing assay

The migration behavior of cells was evaluated using the wound healing assay. After the cell reached 90% confluence, a sterile 200-μL pipet tip was used to scratch three separate wounds through the cells, moving perpendicular to the line. Then, cells were gently rinsed twice with PBS and incubated in serum-free media. At the designated time (0 h, 24 h, 48 h, and 72 h), three randomly selected fields at the lesion border were acquired under a Nikon T1-SM inverted microscope. The number of migrating cells across the wound was counted on the images.

### Transwell migration

Cell migration assays were performed using a transwell system that incorporated a polycarbonate filter membrane with a diameter of 6.5 mm and pore size of 8 μm (Corning) according to the manufacturer’s protocol. The cell suspension (1 × 10^4^) in serum-free culture media was added to the upper chamber, while culture media containing 10% FBS was added to the lower chamber. After 24 h of incubation at 37 °C, the noninvasive cells were removed from the upper chamber by wiping them with cotton-tipped swabs. Cells in the lower chamber were fixed with 4% paraformaldehyde for 30 min and stained with a 0.1% crystal violet solution for 30 min at room temperature. Five fields of adherent cells in each well were randomly photographed and counted under the Nikon T1-SM inverted microscope. The number of cells migrating across the filters was counted on the captured images.

### Statistical analysis

Differences in gene expression between the experimental groups were calculated by analysis of variance (ANOVA), followed by the Bonferroni test. *P* < 0.05 indicated a significant difference, and *P* < 0.01 indicated a highly significant difference.

## Results

### Expression analysis of HGF

**Figure 1:**
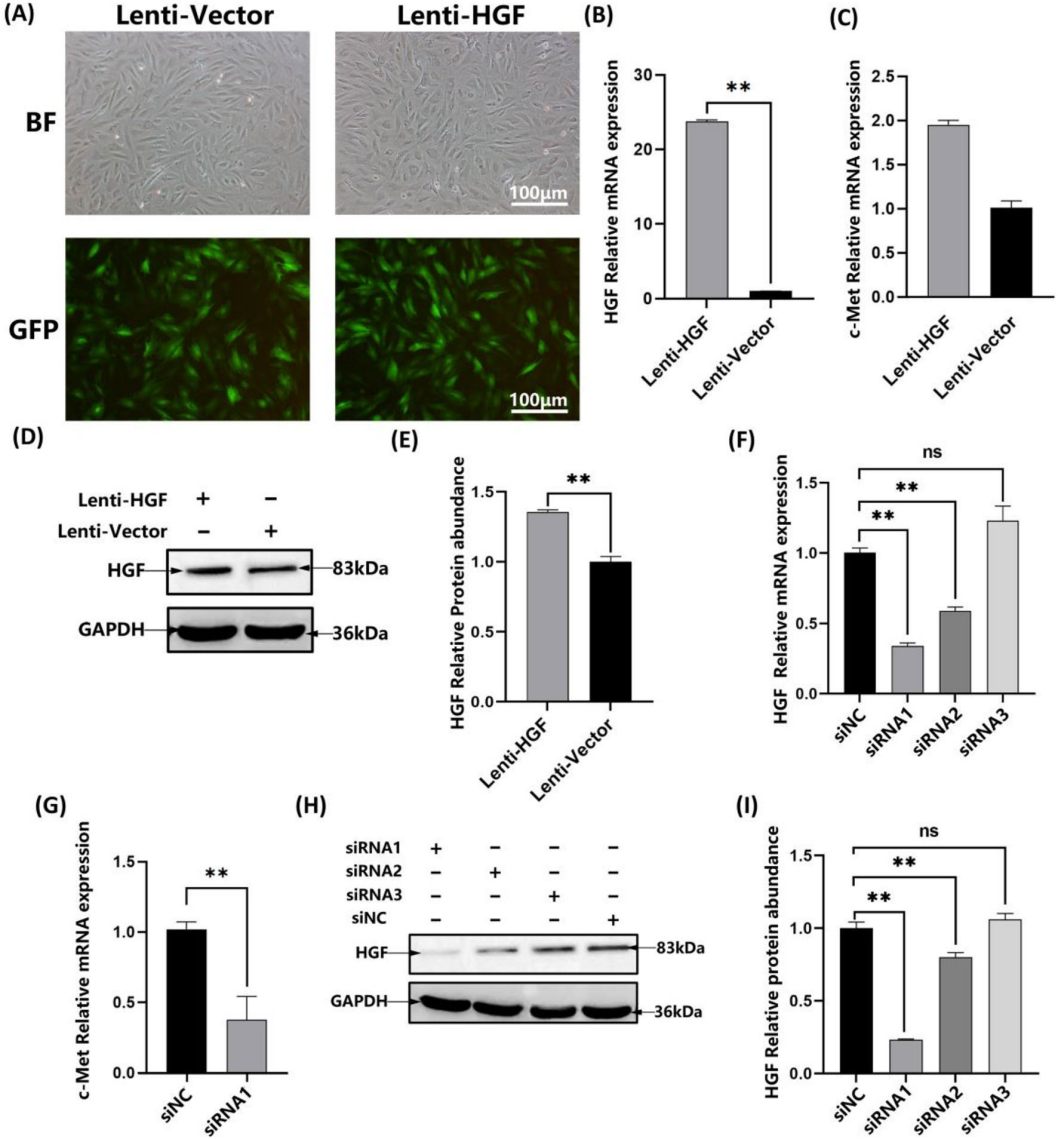
Identification of the efficiency of HGF overexpression and interference in antler MSCs. (**A**) Combined with the bright field (BF) and GFP fluorescence, 90% of antler MSCs were infected by lentivirus from the Vector and HGF groups. Scale bar: 100 μm. (**B**) Relative expression of HGF mRNA in antler MSCs overexpressed by lentivirus. (**C**) Relative expression of c-Met mRNA in antler MSCs overexpressed by lentivirus. (**D**, **E**) Western blot showing overexpression of exogenous HGF in antler MSCs (**F**) Relative expression of HGF mRNA in antler MSCs interfered by siRNA. (**G**) Relative expression of c-Met mRNA in antler MSCs interfered by siRNA. (**H**, **I**) Western blot showed that exogenous HGF was lowly expressed in antler MSCs in the siRNA group. *P < 0.05; **P < 0.01.

Fluorescence microscopy observations revealed that 90% of lentivirus-infected MSCs had GFP fluorescence (Fig. 1A). In the overexpression group, HGF mRNA expression increased to almost 23 times that of the control (Fig. 1B); Western blot further confirmed that HGF was overexpressed in antler MSCs (Fig. 1D, E; full blots in Supplementary Fig. S1). In the siRNA group, its expression was reduced to 66% of the control (empty vector transformation) (Fig. 1F); Western blot further confirmed the low expression of HGF in antler MSCs (Fig. 1H, I; full blots in Supplementary Fig. S1). After overexpression and interference of the *HGF* gene, the changes in mRNA expression of the *c-Met* gene were detected, and the changing trend was the same as that of HGF (Fig. 1C, G).

### Effects of overexpression and interference of HGF on the proliferation of antler MSCs

**Figure 2:**
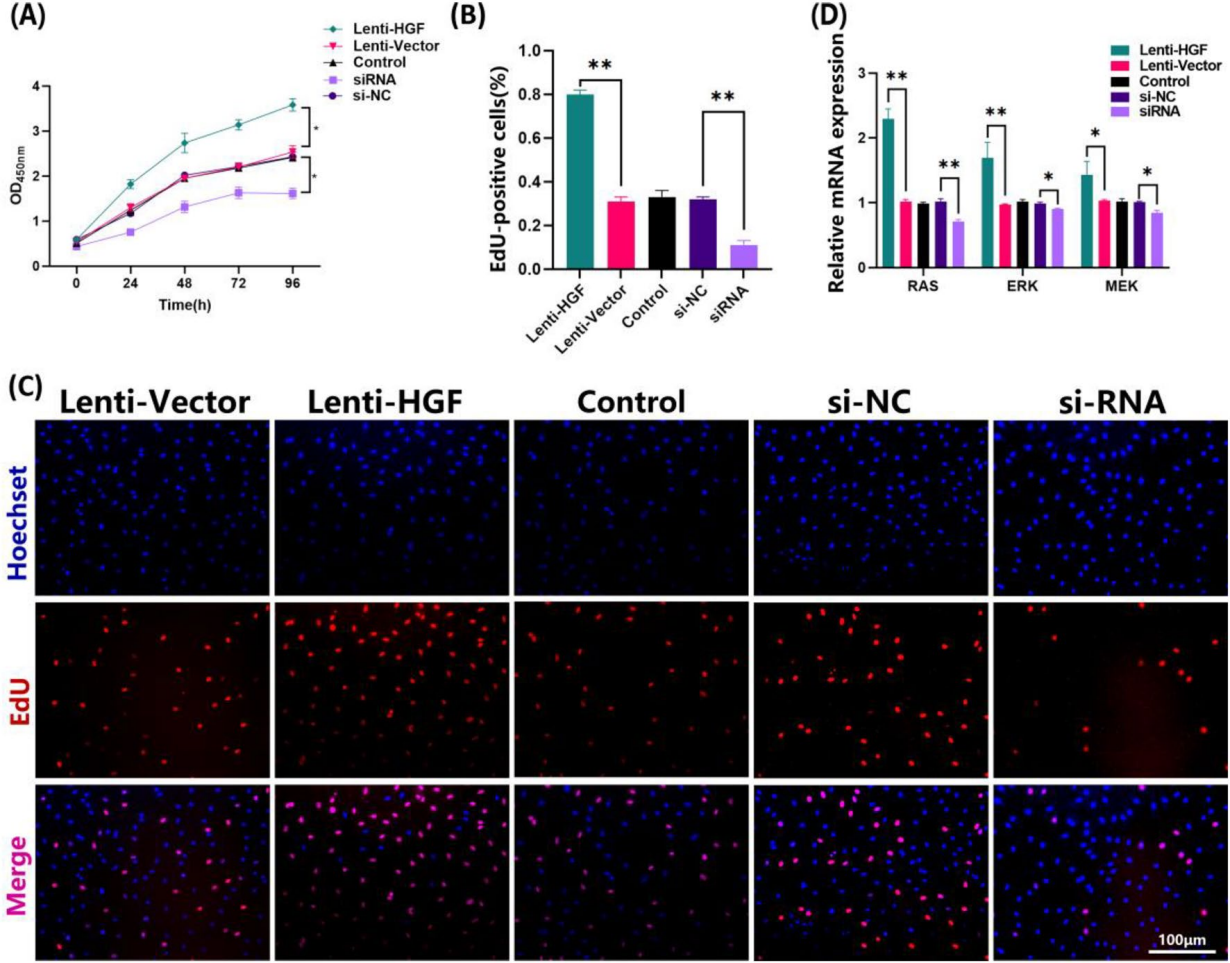
Effect of overexpression and interference with HGF on the proliferation capacity of antler MSCs. (**A**) Viability of antler MSCs detected by CCK8 assay: the Lenti-HGF group was significantly higher than the Lenti-Vector group at each time point (6 h, 12 h, 24 h, 48 h, 72 h, 96 h, and 120 h), and the siRNA group was significantly lower than the NC group at each time point. (**B**) Quantification of the percentage of EdU-positive cells. (**C**) Staining result graph of the proliferation of antler MSCs detected by the EdU method. Scale bar: 100 μm. (**D**) qRT-PCR to detect the relative expression of mRNA of downstream proliferation-related genes *RAS*, *ERK* and *MEK* genes.

CCK8 assays showed higher cell viability in the Lenti-HGF group compared to the Lenti-Vector group, while the siRNA group was significantly lower than the siNC group at any time (*P* < 0.05); there was no significant difference between the three groups Lenti-HGF, si-NC and Control. (*P* > 0.05, Fig. 2A; The raw data of CCK8 assay can be found in Table 1 of the supplementary file). Additionally, the number of EdU-positive cells in the Lenti-HGF group was significantly higher than that in the Lenti-Vector group, while the siRNA group was significantly lower than that in the NC group (*P* < 0.01); there was no significant difference between the three groups Lenti-HGF, si-NC and Control. (*P* > 0.05, Fig. 2B, C; Supplementary Figure 1).

To further investigate the effect of HGF on the proliferation of antler MSCs, the mRNA levels of the downstream proliferation-related genes *RAS*, *ERK* and *MEK* were detected by qRT-PCR (Fig. 2D). The results showed that the mRNA expression levels of *RAS* and *ERK* genes were highly significantly up-regulated (*P* < 0.01) and *MEK* genes were significantly up-regulated (*P* < 0.05) after overexpression of *HGF* genes. Conversely, interfering with *HGF* highly significantly downregulated the mRNA expression of the *RAS* gene (*P* < 0.01), and *ERK* and *MEK* gene mRNA expression was significantly downregulated (*P* < 0.05). These results suggest that overexpression of HGF promotes the proliferation ability of antler MSCs by up-regulating the expression of proliferation-related genes.

### Effects of HGF overexpression and interference on antler MSCs migration

**Figure 3:**
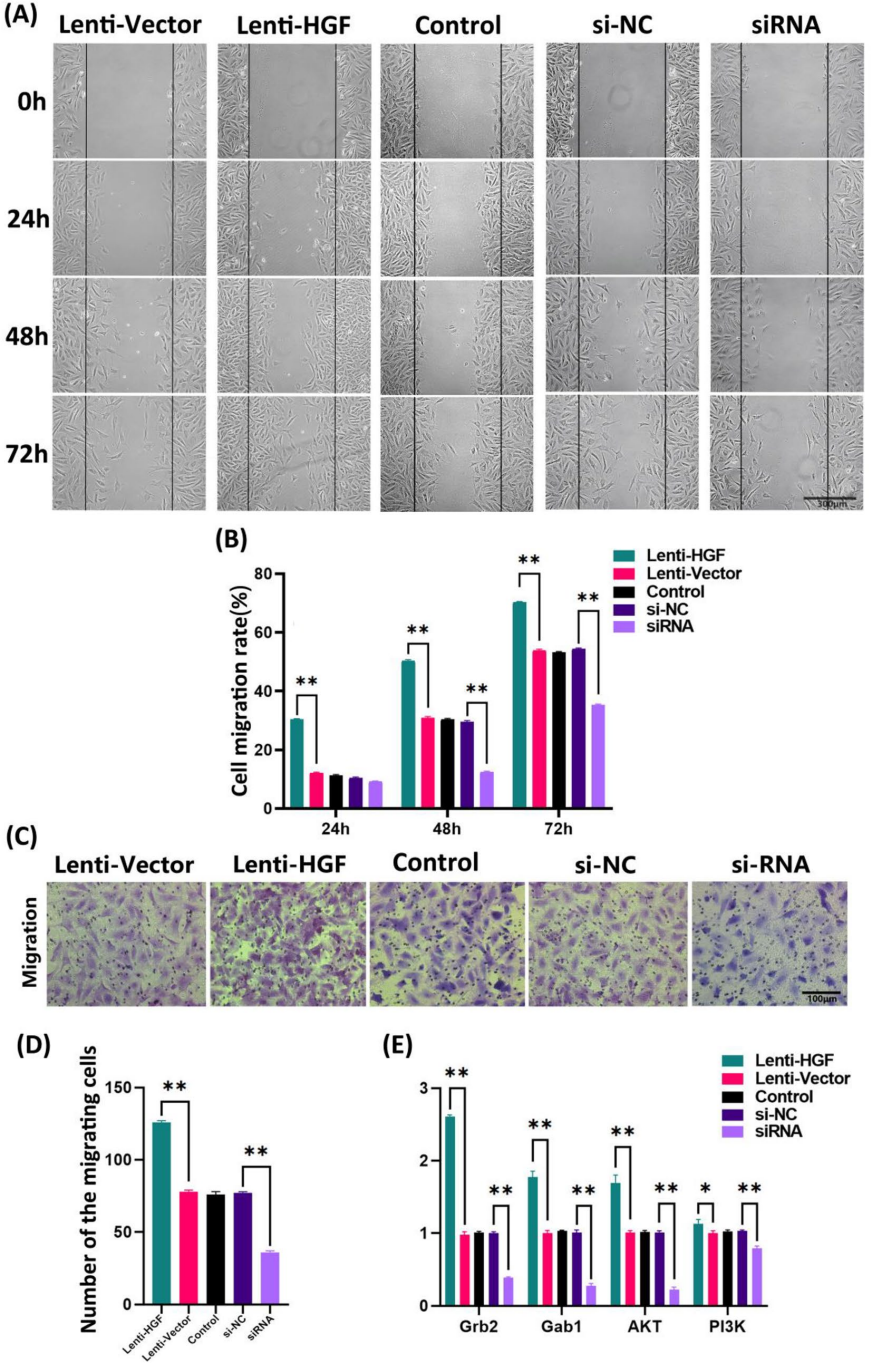
Effect of overexpression and interference with HGF on the migratory capacity of antler MSCs. (**A**, **B**) Wound healing assay to evaluate the migration ability of antler MSCs. (**C**, **D**) Transwell migration assay was performed in antler MSCs. Scale bar: 100 μm. (**E**) qRT-PCR to detect the relative mRNA expression of downstream migration-related genes *Grb1*, *Grb2*, and *AKT* genes .*P < 0.05; **P < 0.01.

A wound healing test was used to examine the effects of overexpression and interference with HGF on the migration of antler MSCs (Fig. 3A; Supplementary Figure 3). The cell healing ability of the Lenti-HGF group was significantly higher than that of the Lenti-Vector and control groups at 24–72 h (*P* < 0.01). The cell healing ability of the siRNA group was not significantly different from that of the si-NC and control groups at 24 h and was significantly lower than that of the si-NC and control groups at 48–72 h (*P* < 0.01). The cell healing ability of the siRNA group was not significantly different from that of the si-NC and Control groups at 24 h and was significantly lower than that of the si-NC and Control groups at 48–72 h (*P* < 0.01). Also, there were no significant differences between the Lenti-Vector and Control groups at any time (*P* > 0.05, Fig. 3B). Transwell test results show that the number of cells passing through the polycarbonate fiber in group Lenti-Vector was extremely significantly higher than the Lenti-Vector and control groups, the cells number of siRNA group was extremely significantly lower than the si-NC and Control groups (*P* < 0.01, Fig. 3C; Supplementary Figure 2), and there was no significant difference between the Lenti-Vector, si-NC and Control groups (*P* > 0.05, Fig. 3D). Transwell detection of migration of antler MSCs was consistent with the wound healing test.

To further investigate the mechanism of the effect of HGF on the migratory activity of antler MSCs, the mRNA level expression of migration-related genes *Grb2*, *Gab1*, *AKT*, and *PI3K* genes were detected using qRT-PCR (Fig. 3E). Overexpression of HGF significantly up-regulated the mRNA level expression of *Grb1*, *Grb2*, and *AKT* genes (*P* < 0.01) and *PI3K* gene expression (*P* < 0.05). Conversely, interfering with HGF highly decreased the expression of *Grb1*, *Grb2*, *AKT* and *PI3K* genes at the mRNA level (*P* < 0.01). These results suggest that overexpression of HGF promotes the migration ability and viability of antler MSCs by up-regulating the expression of migration-related genes.

## Discussion

Previous studies have shown that both tumor suppressor genes and proto-oncogenes in deer are positively selected so that antler MSCs with a significantly higher proliferation rate of cancer cells do not become cancerous during the rapid growth period antler^1,25,26^. HGF/c-Met regulates biological events such as proliferation, migration and morphogenesis of normal cells, but HGF and c-Met are abnormally expressed in many cancers^27^. The binding of HGF to its receptor c-Met can induce dimerization of c-Met and autophosphorylation of tyrosine residues in the carboxy-terminal domain of c-Met, resulting in downstream MAPK, PI3K, RAS and ERK. Also, other signal transduction molecules are sequentially activated, thereby promoting the conduction of downstream signals and participating in the regulation of cell survival, proliferation, and invasion^28^.

The results of this study showed that up-regulation of the *HGF* gene significantly promotes the expression of *c-Met, RAS, MEK, ERK, Grb1, Grb2, AKT,* and *PI3K* genes in antler MSCs, thus significantly promoting the cell proliferation and migration ability of velvet antler MSCs. When c-Met is activated by HGF, PI3K catalyzes the production of phosphatidylinositol-3,4,5 -trisphosphate, which, after Gab1 is coupled to the p85 subunit, eventually leads to the phosphorylation of c-Met^29^. PI3K/AKT pathway is important in regulating cell growth, migration, differentiation and apoptosis^30^. Li et al. performed a 2-DE assay and IPA software differential proteomic analysis of AP and PP cells and found that PI3K/AKT signaling pathway and ERK/MAPK signaling pathway have important roles in both AP and PP cells^31^. Later, Liu et al. found an extremely significant decrease in the proliferation of AP and PP cells by inhibiting the PI3K/AKT signaling pathway using the inhibitor LY294002, which is involved in the developmental regeneration of antlers. When c-Met activation induces binding to the SH2 domain of Grb2, the SH3 domain of Grb2 binds to RAS through SOS, thereby activating RAS^32^. However, the RAS/ERK1/2 pathway is necessary and sufficient for HGF-induced mitogenesis. Kim et al. stimulated ERK1/2 through iPSC-derived iMSC to promote skin cell proliferation^33^. Borowiak et al. found that phosphorylation of ERK1/2 was not detected in c-Met mutant mice when liver regeneration was impaired^34^. When ERK1/2 phosphorylation was inhibited, differentiation of MSCs was significantly induced, and proliferation was inhibited. In contrast, inhibition of Akt activation almost completely eliminated extracellular mineralization^11^. MEK has a major role in proliferation, differentiation, and apoptosis in participating cells^35^. In this experiment, qRT-PCR examination showed that when antler MSCs cells overexpress HGF, the expression of seven genes, i.e., *RAS, MEK, ERK, Gab1, Grb2, AKT,* and *PI3K* increased, which significantly promoted the proliferation and migration ability of antler MSCs (Supplementary Information).

## Conclusion

The migration and proliferation ability of antler MSCs are crucial for antler tissue regeneration^36^. We suggest that HGF/c-Met signaling has an important regulatory role in the migration and proliferation ability of antler MSCs, and the results of this study provide an important theoretical supplement to explore the mechanism of rapid antler growth and regeneration.

## Supplementary Information

Supplementary Information.

## Data Availability

All data generated or analyzed during this study are included in this published article.

## Abbreviations

HGF: Hepatocyte growth factor
MSCs: Mesenchymal stem cells
AnSCs: Antler stem cells
APCs: Antlerogenic periosteum cells
PPCs: Pedicle periosteal cells
RMCs: Reserve mesenchymal cells
IMDM: Iscove’s modified Dulbecco’s medium
c-Met: Cellular-mesenchymal epithelial transition
MEK: Mitogen-activated protein kinase kinase
Gab1: Growth factor receptor-bound protein 1
Grb2: Growth factor receptor-bound protein 2
AKT: Protein kinase B
RAS: Rat Sarcoma Viral Oncogene Homolog
PI3K: Phosphatidylinositol-3-kinase
ERK: Extracellular signal-regulated kinase
iPSC: Induced pluripotent stem cells
iMSC: International Maritime Security Construct
