# Supplementary material for: HGF/c-Met signaling promotes the migration and proliferation of deer antler MSCs
Source: Sci Rep. 2023 Jul 10;13:11121. doi: 10.1038/s41598-023-38116-7 (PMC10333329; doi:10.1038/s41598-023-38116-7)
Supplement: Supplementary file 1 — Supplementary Information. [file 41598_2023_38116_MOESM1_ESM.docx]

**HGF/c-Met signaling promotes the migration and proliferation of deer antler MSCs**

**Miao Wang^1,2^, Chuan Lin^1,2^, Xiaodong Jia^1,2^, Di Fang^1,2^, Qinhua Gao^1,2^, Chunmei Han^1,2*^**

1 College of Animal Science and Technology, Tarim University, Alar 843300, China.

2 Key Laboratory of Tarim Animal Husbandry Science and Technology, Xinjiang Production and Construction Corps, Alar 843300, China.

*Corresponding authors: Chunmei Han,e-mail: chunmeihan224@163.com;Tel:+8617699970211

**Cell recovery**

The cell cryopreservation tube was taken out from the low-temperature environment and quickly put into a 37℃ water bath. After the cell suspension was completely melted, it was transferred into a 2 ml centrifuge tube. Then 1 ml serum-free DMEM medium was slowly added. After centrifugation, the supernatant was discarded. The cell density was adjusted by trypan blue staining, and the cells were inoculated into a culture flask for culture.

**Cell passage**

Passage was performed at 70% confluence. D-Hanks solution was used to wash thrice the cells that were attached to the wall. Trypsin (0.25%) was added to detach the cells. The cells were collected in a 10 mL centrifuge tube and centrifuged at 800 rpm for 5 min. The supernatant was discarded. The cell density was adjusted to 1×10^5^ cells/mL with complete culture medium. The cells (5 mL) were inocu-lated in a flask, and incubated. The medium was completely replaced after 1 day, and then every 3 days.

**Figure S1.** Full-length western blot images of Figure 1D,H.


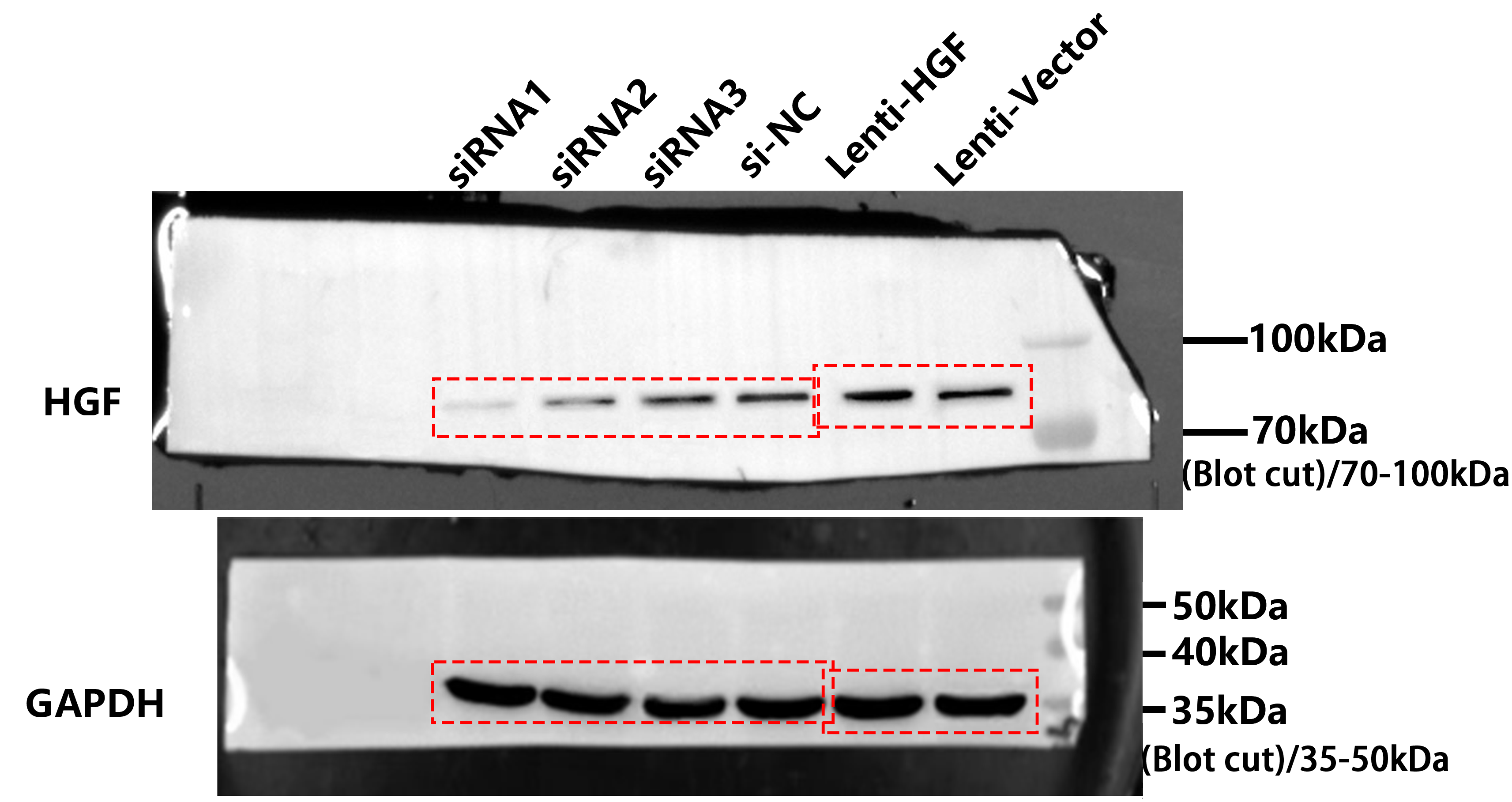


Note: the exposure time is 0.5s.

**Raw data:**

**rTable 1.** **CCK8 assay showed the cell viability in MSCs cells**

|  | Lenti-Vector | | | Lenti-HGF | | | Control | | |
| --- | --- | --- | --- | --- | --- | --- | --- | --- | --- |
| 0h | 0.557 | 0.507 | 0.550 | 0.497 | 0.650 | 0.613 | 0.528 | 0.489 | 0.510 |
| 24h | 1.397 | 1.255 | 1.249 | 1.406 | 1.459 | 1.496 | 1.253 | 1.120 | 1.352 |
| 48h | 1.954 | 1.958 | 1.948 | 2.950 | 2.520 | 2.730 | 2.023 | 1.948 | 1.884 |
| 72h | 2.202 | 2.201 | 2.205 | 3.054 | 3.093 | 3.268 | 2.201 | 2.180 | 2.157 |
| 96h | 2.643 | 2.582 | 2.379 | 3.701 | 3.432 | 3.608 | 2.431 | 2.409 | 2.408 |
|  | si-NC | | | siRNA | | |  | | |
| 0h | 0.562 | 0.596 | 0.611 | 0.511 | 0.554 | 0.506 |  |  |  |
| 24h | 1.120 | 1.104 | 1.105 | 1.080 | 1.043 | 1.010 |  |  |  |
| 48h | 2.051 | 1.996 | 2.004 | 1.228 | 1.460 | 1.254 |  |  |  |
| 72h | 2.185 | 2.303 | 2.146 | 1.581 | 1.530 | 1.770 |  |  |  |
| 96h | 2.545 | 2.381 | 2.363 | 1.614 | 1.728 | 1.503 |  |  |  |

**rFigure 1.** EdU assay assessed the proliferation in Antler MSCs

**
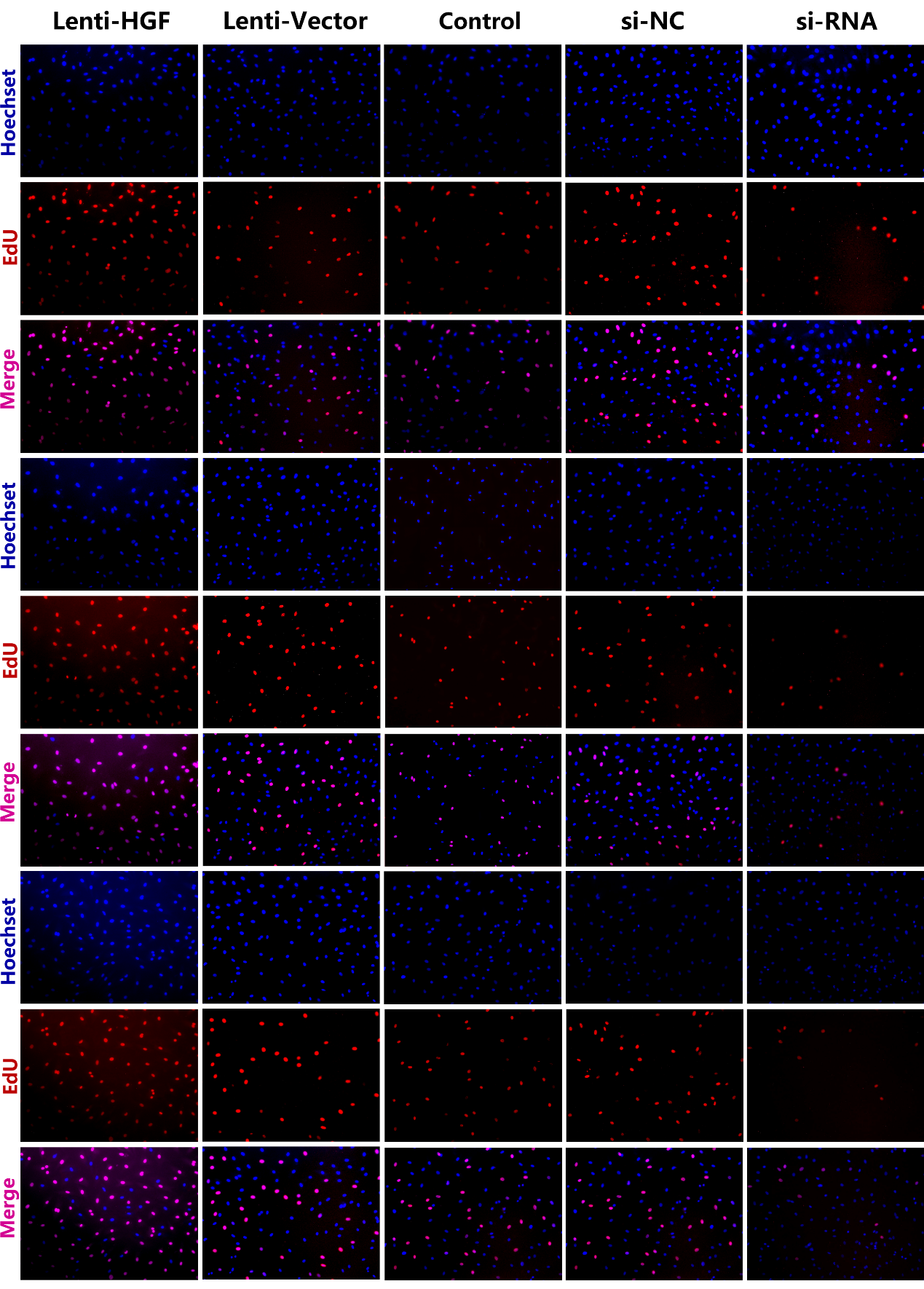
**

**rFigure 2.** Transwell migration assay exhibited the migration ability of Antler MSCs

**
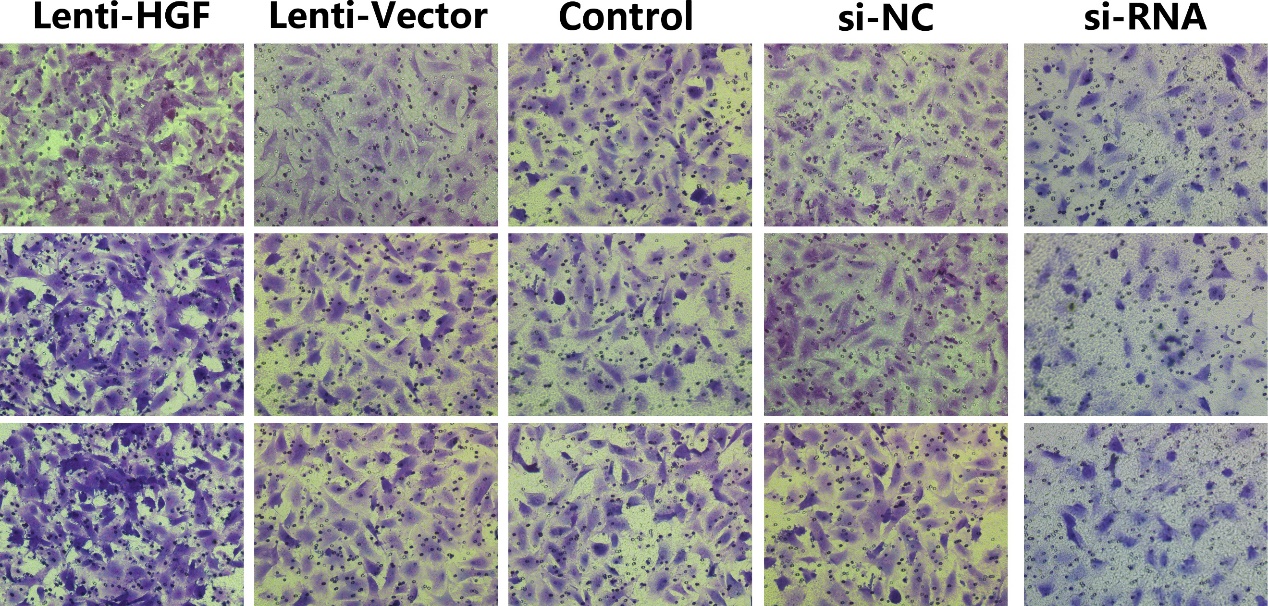
**

**rFigure 3.** Would healing assay showed the migration ability of Antler MSCs

**
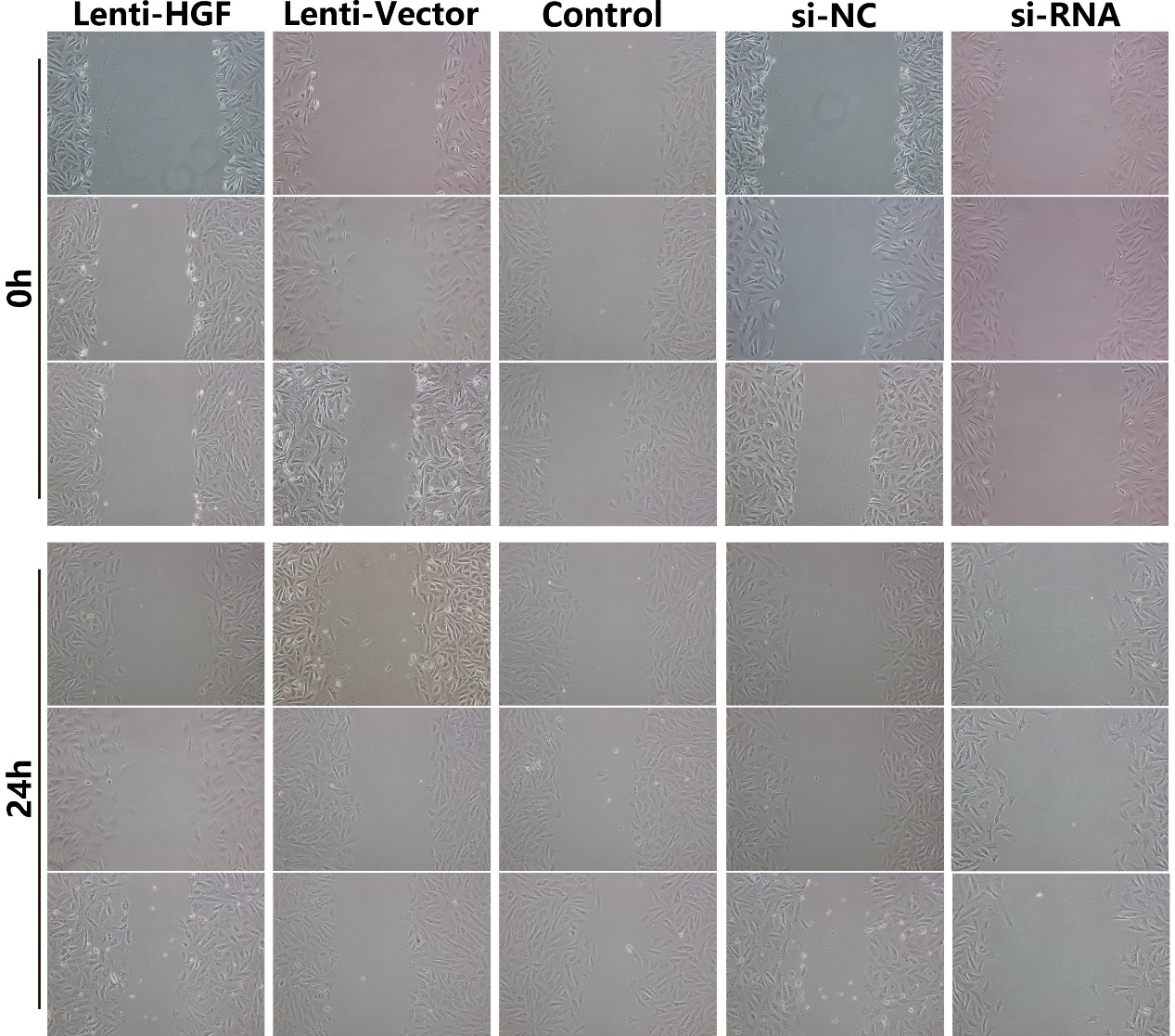

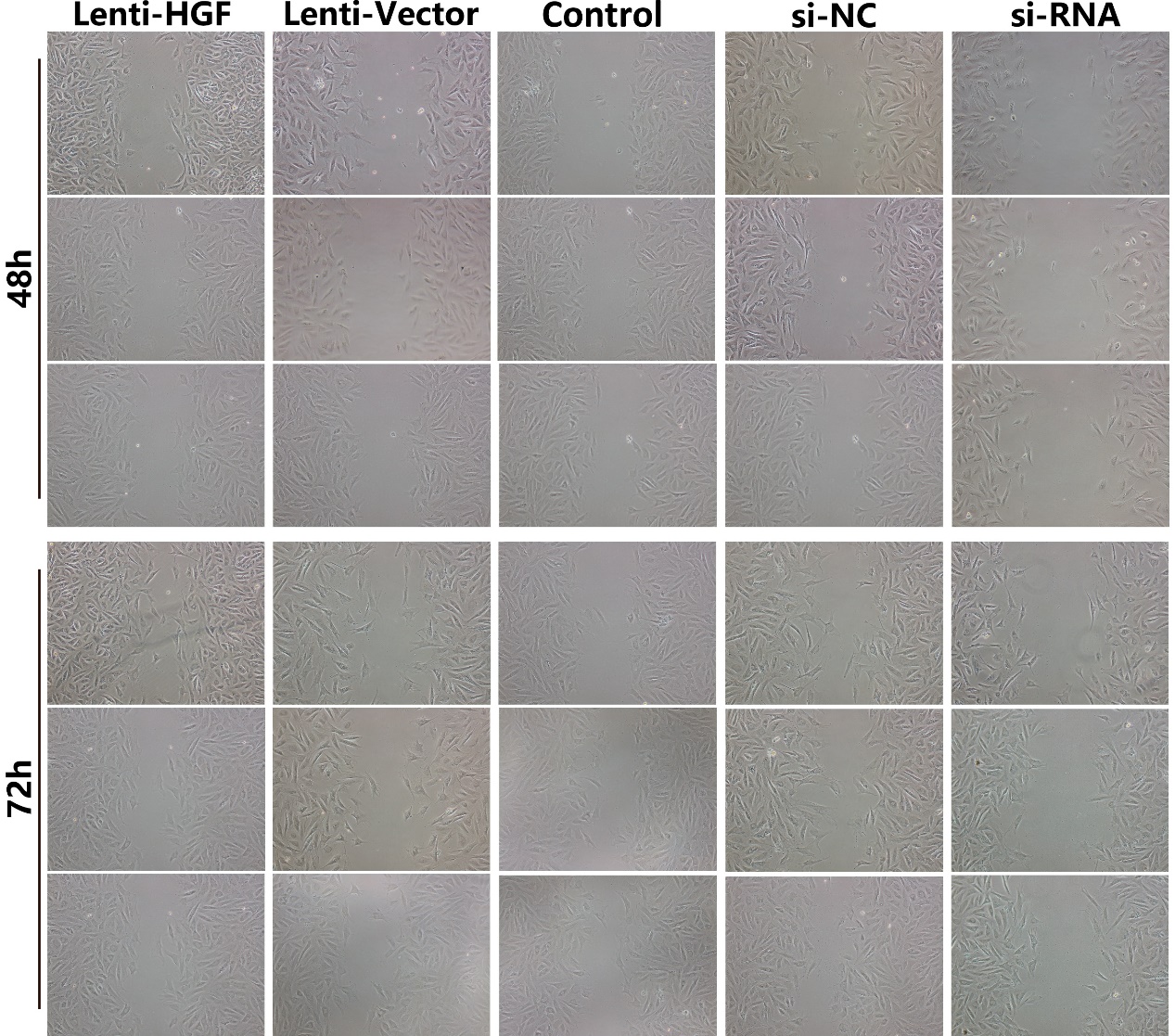
**
